# Progress in rice sheath blight resistance research

**DOI:** 10.3389/fpls.2023.1141697

**Published:** 2023-03-24

**Authors:** Jingsheng Chen, Yuanhu Xuan, Jianghui Yi, Guosheng Xiao, De Peng Yuan, Dandan Li

**Affiliations:** ^1^ College of Biology and Food Engineering, Chongqing Three Gorges University, Wanzhou, China; ^2^ College of Plant Protection, Shenyang Agricultural University, Shenyang, China

**Keywords:** rice sheath blight, resistance, QTL, hormone, nutrition, sugar transporter

## Abstract

Rice sheath blight (ShB) disease poses a major threat to rice yield throughout the world. However, the defense mechanisms against ShB in rice remain largely unknown. ShB resistance is a typical quantitative trait controlled by multiple genes. With the rapid development of molecular methods, many quantitative trait loci (QTLs) related to agronomic traits, biotic and abiotic stresses, and yield have been identified by genome-wide association studies. The interactions between plants and pathogens are controlled by various plant hormone signaling pathways, and the pathways synergistically or antagonistically interact with each other, regulating plant growth and development as well as the defense response. This review summarizes the regulatory effects of hormones including auxin, ethylene, salicylic acid, jasmonic acid, brassinosteroids, gibberellin, abscisic acid, strigolactone, and cytokinin on ShB and the crosstalk between the various hormones. Furthermore, the effects of sugar and nitrogen on rice ShB resistance, as well as information on genes related to ShB resistance in rice and their effects on ShB are also discussed. In summary, this review is a comprehensive description of the QTLs, hormones, nutrition, and other defense-related genes related to ShB in rice. The prospects of targeting the resistance mechanism as a strategy for controlling ShB in rice are also discussed.

## Introduction

Efficient control of crop diseases is a must for sustainable agricultural production (Senapati et al., 2022). Rice is a staple food for half of the world’s population but is severely threatened by diseases. Rice blast, bacterial blight, and rice sheath blight (ShB) are collectively referred to as the three major diseases of rice due to the widespread prevalence resulting in significant yield losses (Liu and Wang, 2016). While rice blast and bacterial blight have been well-controlled through disease resistance breeding based on a deep understanding of the underlying molecular mechanisms of rice resistance to these diseases (Rao et al., 2014), ShB has not. ShB affects the entire life cycle from seedling to heading stage, and causes damage to leaves, sheaths and even panicles, resulting in the wilting of leaves and sheaths and reduced seed setting rates (Savary et al., 1995). The prevention and control of rice ShB follows the conventional strategy of prioritizing prevention followed by comprehensive management, and combines meteorological data collection to monitor rice diseases. The control measures include the breeding of disease-resistant varieties, as well as the application of chemical, biological, or RNA pesticides. As China has no disease-resistant rice varieties, chemical pesticides are mainly used to control rice sheath blight (Rajesh et al., 2016). However, due to the lack of disease-resistant resources of rice germplasm, *Rhizoctonia solani* has high genetic variability and wide host affinity, and can survive from one crop season to the next by forming dormant sclerotia, which increases the difficulty of controlling rice ShB. The resistance of rice to ShB is considered a quantitative trait inheritance controlled by multiple genes. The resistance of different rice varieties to ShB involves multiple quantitative trait loci (QTLs). Identification of QTLs can accelerate the mapping and cloning of resistance genes, which in turn helps to develop rice varieties resistant to ShB (Zuo et al., 2014).

Rice ShB, caused by the fungus *Rhizoctonia solani* Kühn, is one of the three major diseases of rice, causing serious yield losses (Zheng et al., 2013). The sexual state is *Thanatephorus cucumeris*. The pathogen is generally soil-borne and can cause severe disease in a variety of crops (Anderson et al., 2017). Compatible *R. solani* strains can form a fused mycelial network that is genetically highly variable. There are 14 different fusion groups (Anastomosis Group, AG) (Carling et al., 2002a; Carling et al., 2002b), in which 13 groups were named AG1-AG13, and the 14th fusion group AGB1. According to the homology and morphological characteristics of sclerotia, the AG1 strain was further divided into three subgroups: IA, IB and IC. It is generally believed that AG1-IA is the main fusion group causing rice ShB (Singh et al., 2019). AG1-IA infects rice causing ShB in rice-growing regions around the world that can result in up to 50% yield loss (Zheng et al., 2013). The extensive use of nitrogen fertilizer, the introduction of semi-dwarf high-yielding varieties (HYV), and higher crop densities and the resulting moist space are important factors in increasing the incidence of ShB (Savary et al., 1995; Molla et al., 2020). The main rice-producing areas in China are the Central, South China, Southwest, Northeast, North China, and Northwest rice areas which vary in cultivation systems and rice varieties and distributions, as well as the type of rice diseases that occur. In Central and South China, the main disease is ShB, and its incidence area and yield loss are significantly higher than those of rice blast and rice false smut. The yield losses caused by ShB and rice blast in Southwest and North China are similar but significantly higher than that caused by rice false smut (Qi et al., 2021). Therefore, the development of efficient and sustainable ShB control strategies is extremely urgent. The pathogen *R. solani* has a wide host range and high genetic variability. However, to date, few rice varieties that are resistant or immune to ShB have been identified and the development of disease-resistance breeding is therefore limited. Nevertheless, the dissection of the underlying molecular basis of rice resistance to ShB has been of great interest for many years. In recent years, exciting new research on the molecular mechanism of ShB resistance has been emerging. It was found that the WRKY36-*SWEET11* signaling pathway negatively regulates rice ShB and increases the resistance of transformed rice without affecting yield through the interaction of the mesophyll cell-specific mutant SWEET11 and the wild-type (WT) (Gao et al., 2018). Brassinosteroid (BR)-mediated WRKY53 and MPK6 signaling may balance *SWEET2a* expression and thus negatively regulate rice resistance to ShB (Gao et al., 2021). In addition, *AMT1;1*-mediated ${\text{NH}}_{4}^{+}$ transport can accelerate nitrogen metabolism in rice and regulate the expression of subsequent ${\text{NH}}_{4}^{+}$ -dependent ethylene-related genes, thereby promoting rice resistance to ShB and suggesting that appropriate nitrogen uptake and assimilation are necessary for rice defense activation (Wu et al., 2022). In short, the research progress on rice resistance to ShB has developed rapidly.

This review summarizes recent research on the mechanisms of ShB resistance in rice. Since several outstanding reviews about *R. solani*-rice interaction have been recently published (Liu and Wang, 2016; Datta et al., 2017; Molla et al., 2020; Li et al., 2021; Senapati et al., 2022), this review will mainly focus on QTLs, plant hormones, nutrition, and other defense-related genes.

## Quantitative trait loci

In recent years, the identification of broad-spectrum disease resistance genes in rice has become of great interest, including disease resistance gene R, disease resistance regulatory genes, and QTLs (Liu et al., 2021). ShB resistance is a typical quantitative trait controlled by multiple genes (Pinson et al., 2005; Zuo et al., 2014). This is complicated by pleiotropy of major genes and co-dominance of major and minor genes. QTL is a statistically significant association between allele variation at specific loci and the phenotypic traits showing continuous variation (St. Clair, 2010). The identification, mapping, verification, and subsequent characterization of QTLs can accelerate the localization and cloning of important resistance genes. This can aid in the development of ShB-resistant rice varieties. With the increasing use of next-generation DNA sequencing and high-density molecular marker platforms, various QTLs for ShB have been identified and used to determine the source of these traits. QTL mapping has been performed using molecular markers. By analyzing the linkage relationships between the genotype values of molecular markers and the phenotypic values of quantitative traits, QTLs have been mapped to specific sites on chromosomes to estimate their genetic effects (Lynch and Walsh, 1998).

The ShB QTLs have been localized to two major loci: qShB9-2 and qSBR11-1 (Liu et al., 2009; Channamallikarjuna et al., 2010). For the first time, Li et al. (1995) used 113 uniformly distributed RFLP markers to study the F4 mixed population formed by the crosses of the susceptible variety Lemont with resistant variety Teqing, and six QTLs related to ShB resistance were identified (Li et al., 1995). The major qShB9-2 QTL was discovered by Li et al. (1995) and later confirmed in other studies (Han et al., 2002; Pinson et al., 2005; Liu et al., 2013). F2 populations (Pan et al., 1999; Sharma et al., 2009), backcrossed inbred lines (Sato et al., 2004; Eizenga et al., 2015), near-isogenic lines (NILs) (Loan et al., 2004), recombinant inbred lines (RILs) (Pinson et al., 2005), and chromosome segment substitution lines (CSSLs) (Zuo et al., 2013; Zuo et al., 2014) have commonly been used for QTL preliminary mapping. More than 200 QTLs for ShB resistance have been detected in other mapping populations. (Zeng et al., 2015). However, despite the detection of many QTLs for ShB resistance, only qSBR9-2, qSBR11-1, qSB-9^TQ,^ and qSB-11^LE^ were found to be specific genes. Relatively few QTLs contribute to phenotype, and are often affected by agronomic traits such as plant height and heading date. Only the main genes qSB-9^TQ^, qSB-11^LE^ and qSB-11^HJX^ have been used in resistance breeding (Li et al., 2021). There are 26 putative disease-related candidate genes in the qSBR11-1 region, including 11 tandem repeats of chitinase, and 12 candidate genes have also been detected in the qSB-9^Tq^ region (Molla et al., 2020). The major QTL-qSB-9^Tq^ conferring partial resistance to ShB has been verified on chromosome 9 of the Teqing *indica* rice cultivar, and it also has a great potential for enhancing the resistance of *japonica* rice to ShB (Zuo et al., 2008). Pyramiding disease resistance QTLs has been considered as an important strategy to develop ShB resistant cultivars. qSB-11^HJX^, located on chromosome 11 of Huajingxian 74, is one of the most effective resistance QTLs, which can reduce the ShB disease level by about 1.4 at the NIL level (Zhu et al., 2014). By constructing secondary segregation populations and composite interval mapping, qSB-11^HJX^ was mapped between the molecular markers ZY27.49 and ZY27.92-11 with a physical distance of 430 kb (Zhu et al., 2014). Zuo et al. (2013) used CSSL populations in both greenhouse and field environments to fine-locate a QTL (qSB-11LE) of the resistance allele from parent Lemont to the interval of markers Z22-27C and Z23-33C, which was 78.87 kb long. The lines carrying qSB-11^LE^ and qSB-11^HJX^ have a significantly lower level of disease than the recurrent parent and lines with a single QTL under the same genetic background, indicating that QTL pyramiding can further increase the resistance to ShB (Li et al., 2019). The progress of QTL mapping for ShB resistance has been summarized in **Table 1**.

**Table 1:** QTL mapping for ShB resistance in rice.

QTL	chromosome	Marker interval	LOD	Variance explained (%)	Reference
*qSB-1*	1	RG532x	3.8	8	(Pinson et al., 2005)
*qSB-2*	2	C624x	4.3	7	(Pinson et al., 2005)
*qSB-3*	3	R250–C746	6.86	26.5	(Zou et al., 2000)
*qSB-5*	5	Y1049	2.6	6	(Pinson et al., 2005)
*qSB-9*	9	RZ404	3.8	6	(Pinson et al., 2005)
*qShB9-1*	9	RM409-RM257	4.7	5.4	(Liu et al., 2009)
*qShB9-2*	9	RM215-RM245	17.3	24.3	(Liu et al., 2009)
*qSBR-2*	2	RG171-G243A	2.58	11.2	(Yasufumi et al., 2000)
*qSBR-3*	3	G249-G164	2.43	10.5	(Yasufumi et al., 2000)
*qSBR-7*	7	RG511-TCT122	4.34	15.5	(Yasufumi et al., 2000)
*qSBR-11*	11	CT224-CT44	2.75	9.5	(Yasufumi et al., 2000)
*qSBR11-1*	11	sbq11-RM224	4.38	21.59	(Channamallikarjuna et al., 2010)
*qshb1.1*	1	RM151-RM12253	10.7	10.99	(Yadav et al., 2015)
*qshb6.1*	6	RM400-RM253	4.43	13.25	(Yadav et al., 2015)
*qshb7.1*	7	RM81-RM6152	8.8	10.52	(Yadav et al., 2015)
*qshb7.2*	7	RM10-RM21693	6.7	9.72	(Yadav et al., 2015)
*qshb7.3*	7	RM336-RM427	4.12	21.76	(Yadav et al., 2015)
*qshb8.1*	8	RM21792-RM310	4.2	10.52	(Yadav et al., 2015)
*qshb9.1*	9	RM257-RM242	7.9	8.40	(Yadav et al., 2015)
*qshb9.2*	9	RM205-RM105	7.0	19.81	(Yadav et al., 2015)
*qshb9.3*	9	RM24260-RM 3744	3.5	12.58	(Yadav et al., 2015)

With the rapid development of molecular techniques, researchers have used association mapping and genome-wide association studies (GWAS) to identify resistance genes. GWAS are widely used to dissect the broader genetic variability of complex traits in plants (Huang et al., 2010; Zhao et al., 2011; Morris et al., 2013; Liu et al., 2017). Rice germplasm resources are very rich and can provide an excellent natural population for association analyses. To date, GWAS have been used to mine many QTLs related to agronomic traits, biotic and abiotic stresses, and yield in rice (Huang et al., 2010; Famoso et al., 2011; Zhao et al., 2011; Kang et al., 2016). GWAS together with next-generation sequencing are powerful complementary strategies for mapping complex traits in rice. Association mapping can improve the efficiency of aggregation of putative resistance alleles, thereby reducing the cycle of ShB resistance breeding. This strategy also provides a new method for marker-assisted breeding and basic resistance research into ShB (Taguchi-Shiobara et al., 2013; Liu et al., 2021).

## Hormonal signaling

### Auxin

Auxin plays a pivotal role in rice growth and development by regulating virtually all aspects of the plant life cycle (Zhao, 2010). Auxin is the only hormone that can be transported over long distances in plants. *PIN-FORMED 1a* (*OsPIN1a*) is an auxin efflux carrier responsible for auxin polar transport in rice (Xu et al., 2005; Kramer and Bennett, 2006). Sun et al. (2019) inoculated *OsPIN1a* overexpression and RNA-silenced lines with *R. solani*, demonstrating that this gene positively regulates rice resistance to ShB. These results indicated that auxin is correlated with ShB resistance (Sun et al., 2019). Qiao et al. (2020) identified an ShB-responsive small RNA (siR109944), which was inhibited by *R. solani*. Inoculation experiments showed that siR10944 negatively regulates rice resistance to *R. solani*, while its target gene *OsFBL55* (a putative auxin receptor) positively regulates rice resistance to *R. solani* (Qiao et al., 2020).

### Ethylene

As the only gaseous hormone, ethylene is best known for its function in promoting fruit ripening (Bleecker and Kende, 2000). In follow-up studies, ethylene was found to affect plant responses to abiotic and biotic stresses (Broekaert et al., 2006; Kazan, 2015). The results of several studies showed that enhanced ethylene biosynthesis or signal transduction could confer rice broad-spectrum resistance to multiple pathogens (Helliwell et al., 2013; Yang et al., 2017). *OsACS2* encodes a key enzyme for ethylene biosynthesis, the overexpression of which leads to the over-accumulation of ethylene. *OsPBZ1* is a typical *PR* (pathogen-related) gene, and its expression is dramatically induced in response to a pathogen attack. Helliwell et al. (2013) used an *OsPBZ1* promoter to drive *OsACS2* expression in rice. In the absence of pathogens, the expression of *OsACS2* in overexpression lines is similar to that in wild-type plants. However, after pathogen inoculation, both *OsACS2* expression and the ethylene content in *OsACS2*-overexpressing lines were significantly up-regulated, leading to enhanced resistance to ShB, compared with wild-type plants (Helliwell et al., 2013). In addition, our previous work showed that *OsEIL1*, the core component of the rice ethylene signaling pathway which regulates the expression of ethylene-responsive genes, positively regulates rice resistance to ShB (Yuan et al., 2018). These results demonstrate that ethylene contributes to ShB resistance, possibly by activating ROS and phytoalexin production or crosstalk with other defense-related hormones such as jasmonic acid (JA) and salicylic acid (SA) (Yang et al., 2017).

### Salicylic acid and jasmonic acid

While both SA and JA are known defense-related hormones, they differ in function. SA primarily affects plant resistance to biotrophic and hemi-biotrophic pathogens and is critical for system-acquired resistance. JA regulates plant resistance to necrotrophic pathogens and insect herbivory (Howe and Jander, 2008; Browse, 2009; Vlot et al., 2009). Unlike *Arabidopsis*, the SA signaling pathway in rice has two branches. One is the same *NPR1*-mediated pathway while the other is regulated by *OsWRKY45* (Shimono et al., 2007; Yuan et al., 2007). SA was previously thought to be responsible for resistance to biotrophic and semi-biotrophic pathogens. Since *R. solani* was also thought to be a necrotrophic fungus (Vidhyasekaran et al., 1997; Brooks, 2007), the role of SA in rice resistance to ShB remained unclear. Recently, the functional roles of SA in the rice-*R. solani* interaction have been comprehensively investigated. Kouzai et al. (2018) demonstrated that exogenous SA treatment enhanced ShB resistance, while *NahG*-overexpressing rice plants deficient in SA showed increased susceptibility to ShB compared to wild-type plants. These results demonstrated that SA positively regulates rice resistance to ShB (Kouzai et al., 2018). Interestingly, *OsWRKY45* overexpression in rice plants had no positive effects on ShB resistance (Shimono et al., 2012). These results suggest that an *OsWRKY45*-independent SA signaling pathway confers ShB resistance on rice and is most likely *OsNPR1*-related. As a key regulator in plant SA signal transduction, NPR1 is located downstream of SA and upstream of PR protein gene expression. In the npr1 mutant, the gene encoding the PR protein could not be expressed, and the SAR could not be activated to produce disease resistance, indicating that the lack of NPR1 would lead to the loss of SAR in plants (Cao et al., 1994; Cao et al., 1997). In many plants, such as *Arabidopsis*, carrot, rice, tobacco, tomato, wheat, and apple, overexpression of *NPR1* gene can enhance resistance to disease in plants (Fitzgerald et al., 2004; Lin et al., 2004; Chern et al., 2005; Makandar et al., 2006; Malnoy et al., 2007), indicating that NPR1 regulation of the immune response is common in higher plants.

Previous studies found that the gene responsible for JA biosynthesis is essential for rice resistance to ShB, and exogenous application of JA enhanced resistance (Taheri and Tarighi, 2010). Moreover, constitutive expression of the *OsWRKY30* transcription factor promotes JA accumulation and PR gene expression to increase ShB resistance in rice, confirming that JA positively regulates rice resistance to ShB (Peng et al., 2012). Both SA and JA are plant defense-related hormones with sophisticated crosstalk since both synergistic and antagonistic effects have been reported. Surprisingly, based on the above studies, exogenous application of both SA and JA was found to enhance ShB resistance in rice, while suppression of SA and JA significantly reduced resistance (De Vleesschauwer et al., 2013). These results show that the traditional concept of the relationship between SA and JA is not applicable in the rice-*R. solani* system. Therefore, the mechanism of ShB resistance mediated by SA and JA requires further study.

### Brassinosteroids

Brassinosteroids (BRs) are growth-promoting hormones with diverse roles in plant development (Ye et al., 2011). In rice, since BRs regulate plant height, branching, heading date, stress tolerance, and nutrient acquisition, they have become potential targets for breeding improvement (Tong and Chu, 2018; Wang et al., 2020; Yang et al., 2021). Emerging evidence has revealed that BRs also affect the rice response to biotic stresses (Nakashita et al., 2003; He et al., 2017). Taking advantage of rice BR mutants, Yuan et al. (2018) demonstrated that disruption of BR biosynthesis or signal transduction confers ShB resistance in rice, suggesting that BRs are negative regulators of rice resistance to ShB (Yuan et al., 2018). *OsWRKY53*, a newly identified rice BR signal transducer, has been shown to positively regulate BR signaling (Tian et al., 2017). Gao et al. (2021) showed that *OsWRKY53* directly activates the expression of *OsSWEET2a*, a negative regulator of rice resistance to ShB, to confer susceptibility to ShB (Gao et al., 2021). These results provide insight into BR-mediated susceptibility to ShB in rice.

### Other hormones

Current knowledge also implicates hormones related to plant growth and development, such as gibberellin (GA), abscisic acid (Sato et al.), strigolactone (SL), and cytokinin (CTK), either directly or indirectly in plant disease resistance or susceptibility.

GA is a class of plant hormones belonging to the tetracyclic diterpenes, which primarily regulate plant growth and development (Kurosawa, 1926). In rice, local application of GA lowered resistance to semi-living and living nutrients in *Magnaporthe oryzae* and *Xanthomonas oryzae* pv. *oryzae* (*Xoo*) (Yang et al., 2008; Qin et al., 2013). Although the means by which GA influences innate immunity in rice is not fully understood, several studies have demonstrated that GA is also associated with the inhibition of defense-related gene expression, plant antitoxin biosynthesis, and the regulation of SA and JA levels (Tanaka et al., 2006; Yang et al., 2008; Qin et al., 2013). GA is generally considered a negative regulator of rice innate immunity. SLENDER RICE 1 (SLR1) is the only DELLA protein in rice that inhibits GA signaling (Ikeda et al., 2001) and its mutation significantly increases susceptibility to *Xoo* (Yang, 2009). In addition, GA antagonizes JA signaling *via* DELLA proteins during rice development and immunity, thus acting as a major regulator of both hormonal pathways (Ikeda et al., 2001; Navarro et al., 2008).

ABA regulates many physiological processes involved in growth and development. Specifically, ABA has been extensively studied for its role in resisting abiotic stresses such as high salinity, drought, and low temperatures (Cutler et al., 2010). In recent years, ABA has also been shown to be significantly involved in the regulation and integration of defense responses. Both positive and negative ABA effects on disease resistance have been previously reported. However, ABA is primarily a negative regulator of immunity, regulating rice resistance to *Xoo* and *M. oryzae* (Jiang et al., 2010; Xu et al., 2013; Cao et al., 2016).

SLs are hormones found in many plants that inhibit branching and are involved in various developmental processes. The number of tillers, a key goal in rice breeding (Wang et al., 2018), can be significantly altered by genetically or chemically modifying the SL pathway (Waters et al., 2017). SLs stimulate seed germination of parasitic plants, induce the branching of mycorrhizal hyphae, and inhibit the branching in plants (Kumar et al., 2015; Kameoka and Kyozuka, 2018; Wang et al., 2020; Bhoi et al., 2021; Mashiguchi et al., 2021). However, since few studies to date have explored the role of SLs in resistance to ShB, this area of research requires further investigation.

**Figure 1:**
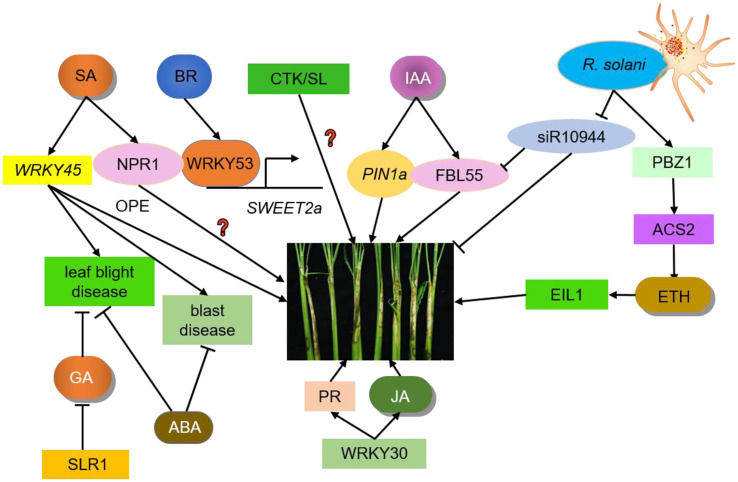
Crosstalk between hormones and ShB. IAA, ETH, SA, JA, BR, GA, ABA, CTK, and SL regulate ShB resistance. PIN1a is an auxin efflux carrier responsible for auxin polar transport in rice. PIN1a positively regulates rice resistance to ShB. siR109944 expression is suppressed by *R. solani* inoculation. ACS2 leads to over-accumulation of ethylene. PBZ1 expression level is significantly induced in response to pathogen attacks. Following pathogen inoculation, the ACS2 levels and ethylene contents in ACS2-overexpression lines are significantly up-regulated, resulting in enhanced resistance to ShB. EIL1, the core component of the rice ethylene signaling pathway which regulates the expression of ethylene-responsive genes, positively regulating rice resistance to ShB. The SA signaling pathway in rice has two branches, one is the same NPR1-mediated pathway while the other is regulated by WRKY45. WRKY45 overexpression rice plants results in resistance to blast disease and leaf blight disease but has no positive effects (OPE) on ShB resistance. The function of NPR1 in the rice-ShB interaction remains unknown. Constitutive expression of transcription factor WRKY30 promotes JA accumulation and PR gene expression to increase ShB resistance in rice. JA positively regulates rice resistance to ShB. BR is a negative regulator of rice resistance to ShB. WRKY53 directly activates the expression of SWEET2a, a negative regulator of rice resistance to ShB, to confer susceptibility to ShB. SLR1 is the only DELLA protein in rice that inhibits GA signaling and its mutation significantly increases the disease susceptibility to leaf blight disease. ABA is primarily a negative regulator of immunity that regulates rice resistance to leaf blight disease and blast disease. The effects of SLs and CTK on the ShB process are not clear and require further investigation.

CTK is the developmental hormone related to plant immunity. CTK is the earliest plant hormone found in maize seeds that can promote cell division. *M. oryzae* increases the CTK content of the host to facilitate its infection and rice can utilize this increase as a signal for pathogen infection to activate the defense response (Jiang et al., 2013). In *Arabidopsis*, high concentrations of CTK increase SA-mediated resistance to biotrophic pathogens, while lower concentrations increase sensitivity to biotrophic pathogens (Choi et al., 2010; Argueso et al., 2012). However, studies investigating the effects of GA, ABA, SLs, and CTK on rice resistance have primarily focused on rice blast and rice bacterial blight. The effects of these hormones on the ShB process are not clear and require further investigation. Plant hormones do not act independently on pathogens, but instead resist infection *via* mutual antagonism or synergy with other hormones (**Figure 1**). This interaction or crosstalk between individual hormones is thought to enable plants to adjust their induced defense arsenals based on the type of infective agent and cost-effectively use their limited resources (Verhage et al., 2010; De Vleesschauwer et al., 2013).

## Nutrition

The primary purpose of pathogenic fungal infection of plants is to obtain nutrients for survival. Fungi use plants as carbon and nitrogen sources, which are crucial for the growth and development of plants themselves. Therefore, the nutritional status of rice determines the resistance of rice to ShB disease. Next, the effects of two plant nutrients, sugar and nitrogen, on rice ShB resistance are summarized.

### Sugar

**Figure 2:**
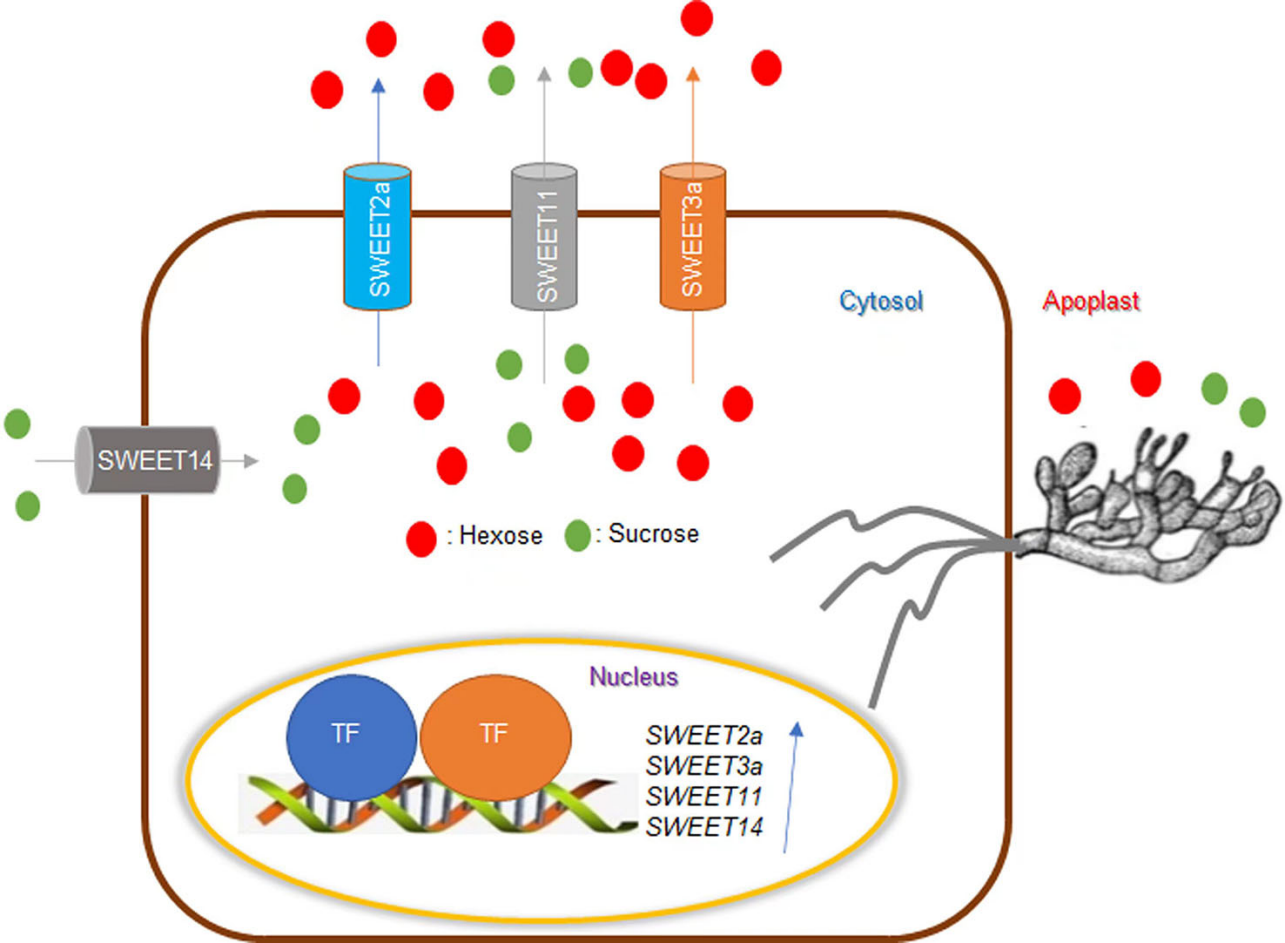
Effects of sugar on rice ShB. SWEET transports hexose and sucrose across the membrane. *SWEET11*/*Os8N3* plays a vital role in seed filling and can also be exploited by pathogens to transport sugar to the extracellular space to provide nutrients for pathogens. While the *sweet11* mutant showed resistance to multiple pathogens, it is defective in seed filling, thus reducing yield. *Rhizoctonia solani* AG1-IA effector AOS2 is secreted and targeted in the nucleus to interact with *WRKY53* and GT1 to activate *SWEET2a* and *SWEET3a* to efflux sugar for nutrition. *SWEET2a* and *SWEET11* negatively regulate ShB resistance in rice. Alternatively, *SWEET14* contributes to ShB resistance in rice. Sugar transporters are extensively involved in the interaction between rice and ShB.

The assimilation products of photosynthesis in plants are transported in the form of sugars. Sugars from host plants are known to be taken up by fungi (Aked and Hall, 1993; Sutton et al., 1999). Currently, there are two hypotheses to explain the role of sugars in plant-pathogen interactions. The first is the “pathogen starvation hypothesis” and the second is the “sugar signaling hypothesis” (Bezrutczyk et al., 2018). Based on this inference, Gao et al. (2018) introduced a dominant-negative version of *OsSWEET11* that is driven by the *rubisco* promoter which is expressed in green tissues but not in seeds to create ShB-resistant rice without penalty to yield (Gao et al., 2018). Sugar Will Eventually be Exported Transporter (SWEET) proteins transport hexose and sucrose across the cell membrane (Chen et al., 2015). *OsSWEET11*/*Os8N3* play a vital role in seed filling and can also be exploited by pathogens to transport sugar into the extracellular space to provide nutrients for the pathogens. The *Ossweet11* mutant shows resistance to multiple pathogens, but its seed-filling defect has a negative impact on the yield (Chen et al., 2010; Bezrutczyk et al., 2018). Therefore, the implantation of mutated OsSWEET11 in green tissues to inhibit the function of endogenous OsSWEET11 achieves enhanced disease resistance without compromising the yield. Gao et al (2021) revealed the important role of sugar in the rice-ShB interaction and confirmed that manipulation of endogenous sugar levels can alter rice susceptibility to ShB. Additional evidence is that *OsSWEET2a* negatively regulates rice resistance to ShB (Gao et al., 2021). Furthermore, the *R. solani* AG1-IA effector AOS2 that is secreted and targeted in the nucleus, interacts with WRKY53 and grassy tiller 1 (GT1) to activate *SWEET2a* and *SWEET3a* resulting in sugar efflux for nutrition (Yang et al., 2022). In addition, a recent study identified new QTLs for ShB resistance which included *OsSWEET13* and *14* (Li et al., 2022). These results were consistent with Kim et al. (2021) study that identified *OsSWEET14* overexpression plants that exhibited ShB resistance, while *Ossweet14* mutants were more susceptible compared to wild-type plants, demonstrating that *OsSWEET14* contributes to ShB resistance in rice. In summary, these studies indicate that sugar transporters are extensively involved in the interaction between rice and ShB (**Figure 2**).

### Nitrogen

**Figure 3:**
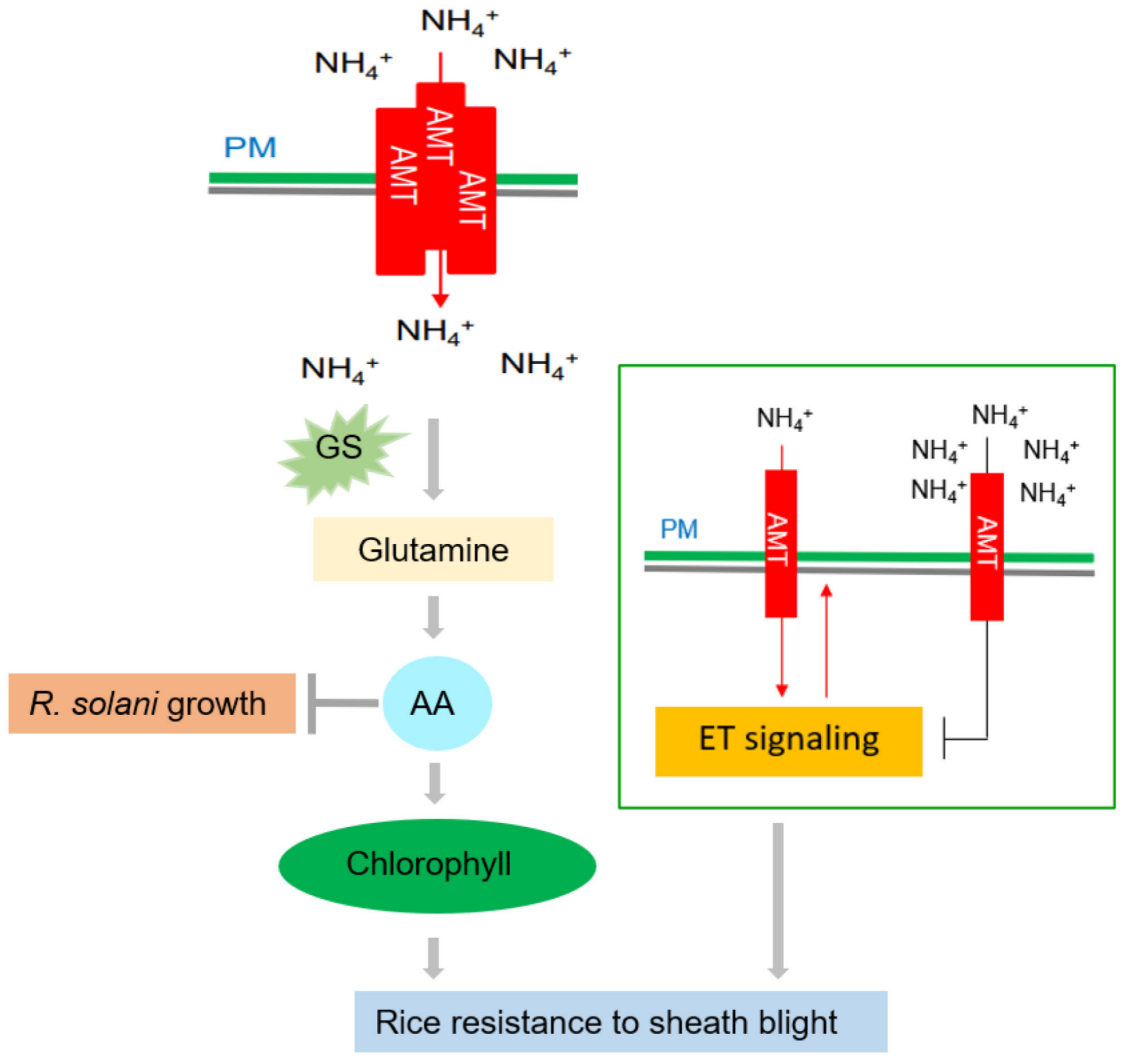
Effects of nitrogen on rice ShB. The rice ammonium transporter *AMT1;1* positively regulates rice resistance to ShB. This phenomenon is caused not by ammonium itself, but by N-derived metabolites. *AMT1;1* enhances the resistance of rice to ShB by promoting the accumulation of N metabolites, such as amino acids and chlorophyll, and activating the downstream ETH signaling pathway. Amino acid (AA) accumulation can inhibit *R. solani* and promote chlorophyll synthesis, which is a positive regulator of rice ShB. A low concentration of ${\text{NH}}_{4}^{+}$ activates the ETH signal through AMT and a high concentration of ${\text{NH}}_{4}^{+}$ inhibits the ETH signal. ETH signaling positively regulates ShB resistance and ${\text{NH}}_{4}^{+}$ uptake, suggesting that ETH signaling acts downstream of AMT and that ${\text{NH}}_{4}^{+}$ uptake is also under feedback control.

Since the Green Revolution in the 1960s, nitrogen (N) fertilizer has played a significant role in increased rice yields. This issue originated when the *semi-dwarf1* (*sd1*) allele was used extensively in rice breeding to develop semi-dwarf Green Revolution varieties (GRVs) that enhanced lodging resistance and increased yield. However, these GRVs exhibited poor nitrogen use efficiency (NUE) and therefore, large amounts of N fertilizer were necessary to produce the desired high yield in these varieties (Liu et al., 2022). High N fertilizer input promotes rice growth and development and also guarantees yield, but excessive N fertilizer will increase the prevalence of ShB (Savary et al., 1995). There is thus a dilemma where high applications of N fertilizer ensure good GRV yields but concomitantly aggravate the prevalence of ShB. Hence, it is particularly important to explore the N-mediated mechanism of rice resistance to ShB. A recent study reported clarification of the mechanism of ammonium-mediated resistance to ShB (**Figure 3**). Beginning with a susceptible mutant, Wu et al. (2022) found that the rice ammonium transporter OsAMT1;1 positively regulates rice resistance to ShB. However, this phenomenon was caused not by ammonium itself, but by N-derived metabolites (e.g., amino acids). Combining the results of genetics with physiological and biochemical experiments, Wu et al. (2022) proposed that OsAMT1;1 enhanced rice resistance to ShB *via* the accumulation of N metabolites (such as amino acids and chlorophyll) and activation of the downstream ethylene signaling pathway (Wu et al., 2022). In summary, overexpression of *OsAMT1;1* could simultaneously improve yield (Ranathunge et al., 2014) and resistance to ShB. This suggests that *OsAMT1;1* is a promising target for the genetic improvement of rice. Sun et al. (2014) identified and cloned a rice NUE-related QTL (*qNGR9*) and found that it is synonymous with *DENSE AND ERECT PANICLE 1* (*OsDEP1*). Rice varieties carrying *dep1* alleles are insensitive to N supply and display increased NUE (Sun et al., 2014). Meanwhile, *OsDEP1* was also reported to affect ShB resistance. *OsDEP1*-silenced plants and *Osdep1* mutants are resistant to ShB, while *OsDEP1* overexpression lines demonstrated increased susceptibility to ShB. OsDEP1 interacts with the transcription factor LPA1 (Loose Plant Architecture 1) to inhibit its function of activating the expression of *OsPIN1a*, thereby inhibiting rice resistance to ShB (Miao Liu et al., 2021).

## Other defense-related genes

Due to the lack of ShB-resistant rice germplasm resources, it is challenging to breed varieties resistant to ShB using traditional breeding methods. The use of genetic engineering technology to transform ShB resistance-related genes is one of the most effective means to develop resistant varieties. In recent years, researchers have isolated and identified many ShB resistance-related genes from rice. IDD14 and IDD13 activate *PIN1a* to promote rice resistance to ShB (Sun et al., 2019; Sun et al., 2020). The interaction between *DEP1* and *IDD14* negatively regulates rice defense against ShB (Liu et al., 2021). Chitin is one of the main components of the fungal cell wall and the *chitinase* (*chi11*) gene can enhance ShB resistance in rice (Baisakh et al., 2001; Datta et al., 2001). Lignin is an important component of the structural integrity of plant cell walls and its deposition enables plant cell walls to resist pathogen infection. Furthermore, some phenols and free radicals produced during lignin synthesis can reduce the infection ability of pathogens by affecting the activity of physiologically-related enzymes of pathogens (Chezem et al., 2017). A GWAS in maize has shown that the F-box protein ZmFBL41 interacts with and degrades ZmCAD (lignin biosynthesis enzyme) to inhibit ShB resistance (Li et al., 2019). *WRKY* transcription factors have been extensively studied. Studies have demonstrated that they play key roles as regulators in plant immune responses under a variety of biotic stresses (Cui et al., 2019). *WRKY* genes are crucial in inhibiting or activating both plant defense responses *via* direct or indirect interaction with PAMPs/effector proteins or *via* MAPK regulation (Phukan et al., 2016). Multiple *WRKY* transcription factors regulate rice resistance to ShB through transcriptional activation or repression. The transcription factors *WRKY24* and *WRKY70* are highly expressed in disease-resistant rice varieties (Zhang et al., 2017). Previous studies have shown that transcription factors such as *OsWRKY4,13,30*, and *80* enhance ShB resistance in rice (Wang et al., 2015; Peng et al., 2016; Lilly and Subramanian, 2019). However, *OsWRKY53-*overexpression lines are more susceptible to ShB (Yuan et al., 2020). The rice sugar transporters *SWEET11* and *14* negatively and positively regulate rice resistance to ShB, respectively (Gao et al., 2018; Kim et al., 2021). In addition, DOF11 promotes rice resistance to ShB *via* direct activation of *SWEET14* (Kim et al., 2021).

## Conclusion and perspectives

Rice is an important food crop throughout the world. However, it is susceptible to diseases such as ShB. The pathogen *R. solani* has a wide host range and can infect more than 200 plant species. These host plants belong to the Poaceae, Fabaceae, Solanaceae, Amaranthaceae, Brassicaceae, Rubiaceae, Malvaceae, Asteraceae, Araceae, Moraceae, and Linaceae families (Chahal et al., 2003). Up to 188 plant species belonging to 32 families were found to be infected by this fungus in Japan (Kozaka, 1961). In India, 62 important economic plants and 20 weed families have been reported (Roy, 1993). Several weed plants have been identified as adjunct hosts of pathogens in the absence of rice plants (Acharya and Sengupta, 1998) and as inoculums that contribute to the further spread of disease. In recent years, the prevalence of ShB has increased due to the increased intensity of climate change, the promotion and planting of dwarf varieties and hybrid rice, and the intensive rice production system characterized by the large-scale application of nitrogen fertilizer, high planting density, and wide use of high-yield varieties in the process of cultivation and management, seriously affecting both rice yield and quality (Slaton et al., 2003; Molla et al., 2020).

Therefore, it is essential to study ShB to contribute to the understanding and prevention of this severe disease. The planting of ShB-resistant varieties is the most economical and effective way to control ShB. Excavating ShB resistance germplasm resources and mapping ShB resistance genes are the premise and basis of the breeding of resistant varieties. The rice yield loss caused by ShB is estimated to be 10-40% per year and is becoming a major threat to rice cultivation (Savary et al., 2000). However, breeders have not yet identified highly resistant or immune varieties, significantly restricting the development of ShB resistance breeding and the discovery of excellent resistance genes. In the process of green and high-quality agricultural development in China, we should also increase the integrated promotion of green prevention and control technology of rice diseases and insect pests, and strive to make breakthroughs in the breeding and promotion of disease-resistant varieties, the screening and development of new pesticides such as biological and RNA pesticides, and the efficient application of pesticide technology. To date, there are many reported QTLs associated with ShB resistance. However, only a few genes have been shown to regulate ShB resistance. Therefore, the mining and screening of resistant germplasm resources and the mapping of ShB resistance genes/QTLs remain significant topics for future research on ShB.

When plants encounter biotic stresses, plant hormones activate defense genes to coordinate effective defense responses. The pathways regulated by SA and JA constitute the key part of the regulation of the immune system hormones (Wasternack and Song, 2017; Zhang and Li, 2019). Plant hormone signal transduction pathways can regulate the defense response of rice against *R. solani*. Transcription factors (such as WRKY, MYB, and RAV) are a class of proteins that regulate gene expression and are usually involved in different plant hormonal signaling pathways (Yamasaki et al., 2005; Yamasaki et al., 2008; Singh and Subramanian, 2017; Yuan et al., 2018). They bind to the promoter region upstream of the target gene to activate or inhibit the expression of the target gene and stimulate the defense mechanism of rice against *R. solani*.

*SWEET* is a gene family widely distributed in prokaryotes, animals, and many members are found in plants (especially in higher vascular plants). Plant *SWEET* genes have diverse functions, affecting the reproductive development of plants, participating in phloem sugar loading, pollen development, fruit or seed development, nectar secretion, leaf senescence, ion transport, and other physiological processes, including plant-pathogen interaction and abiotic stress (Yang et al., 2006; Seo et al., 2011; Chen et al., 2012; Lin et al., 2014; Sosso et al., 2015). Plant-pathogen interaction is a complex relationship determined by a variety of factors. Pathogens secrete transcription activator-like effectors (TALEs) into host cells and act as transcriptional activators of plant target genes in host cells to facilitate pathogen reproduction or interfere with innate plant immunity (Tadege et al., 1998; Asai et al., 2016), while sugars provide a carbon source for pathogens and their host plants and the sugar signal can therefore induce the expression of defense genes. *SWEET* sugar transporters play a key role in regulating the redistribution of sucrose in plant tissues, suggesting that they are vital for balancing resistance and yield. To simultaneously improve yield and resistance, Li et al. (2012) used a miRNA to specifically inhibit the expression of *OsSWEET11* in rice leaf tissues, thereby increasing resistance to bacterial blight while maintaining the seed setting rate. Photosynthetic tissues in plants are the main source of sugars, which are then transported across the membrane into the phloem under the mediation of sugar transporters. This process provides a pathway for sugars to enter various sugar-dependent tissues and cells (Bezrutczyk et al., 2018). Therefore, *SWEET* genes can positively or negatively regulate rice resistance to ShB. As a new member of the sugar transporter family, SWEET provides a channel for pathogens to hijack sugar from hosts. The separation of disease-resistant varieties and disease-resistant genes is of great significance for future breeding. Therefore, these SWEET-associated molecular mechanisms contributing to rice resistance to ShB should be further explored.

Nitrogen is an important mineral nutrient for plant growth and development. It is also an important component of nucleic acids, chloroplasts, proteins, and many secondary metabolites. Increased nitrogen fertilizer application is the major means of increasing crop yield. Improving NUE is the common goal of many researchers. Previous studies have shown that high doses of N fertilizers can lead to a significant increase in ShB incidence (Molla et al., 2020). However, a limited nitrogen supply will limit plant growth and yield. Under the condition of limited nitrogen fertilizer, *AMT1;1*-mediated ${\text{NH}}_{4}^{+}$ transport can accelerate nitrogen metabolism in rice and regulate the expression of subsequent ${\text{NH}}_{4}^{+}$ -dependent ethylene-related genes, thereby promoting ShB resistance. It has been suggested that adequate nitrogen uptake and assimilation are essential for the activation of rice defense mechanisms (Wu et al., 2022). With the advances in genomic technology and functional genomics research, the underlying genetic mechanisms responsible for plant nitrogen uptake, utilization, and signal regulation can be extensively analyzed, providing a theoretical and technical foundation for improving crop NUE *via* molecular genetic means.

Rice is a major food crop throughout the world and its safe production is of great significance in solving the global food crisis. ShB is the main factor responsible for reducing rice yield. In addition to the use of good farming systems, the use of chemicals is one of the main ways to control disease, but it increases costs and pollutes the environment. This review summarizes the research progress in ShB based on studies on QTLs, hormones, nutrition, and defense-associated genes, analyzes the mechanism of rice resistance to ShB, and provides a comprehensive and systematic theoretical basis for the future breeding of ShB-resistant varieties of rice.

## Author contributions

DDL, DPY, and JSC designed this work, JSC, YHX, JGY, GSX, DPY and DDL wrote the manuscript. All authors contributed to the article and approved the submitted version.
