# Corrigendum: Progress in rice sheath blight resistance research

**DOI:** 10.3389/fpls.2023.1232679

**Published:** 2023-07-13

**Authors:** Jingsheng Chen, Yuanhu Xuan, Jianghui Yi, Guosheng Xiao, De Peng Yuan, Dandan Li

**Affiliations:** ^1^College of Biology and Food Engineering, Chongqing Three Gorges University, Wanzhou, China; ^2^College of Plant Protection, Shenyang Agricultural University, Shenyang, China

**Keywords:** rice sheath blight, resistance, QTL, hormone, nutrition, sugar transporter

In the published article, there was an error in **Figures 1**, **3**. **Figures 1**, **3** images were reversed, but the figure legends were correct.

**Figure 1:**
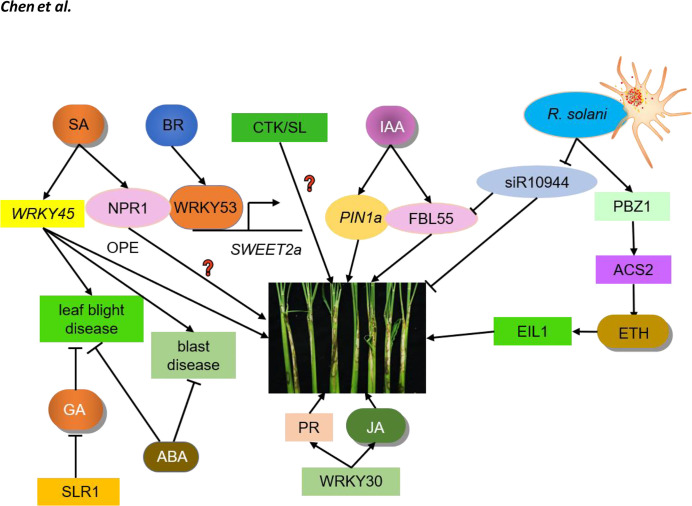
Crosstalk between hormones and ShB. IAA, ETH, SA, JA, BR, GA, ABA, CTK, and SL regulate ShB resistance. PIN1a is an auxin efflux carrier responsible for auxin polar transport in rice. PIN1a positively regulates rice resistance to ShB. siR109944 expression is suppressed by R. solani inoculation. ACS2 leads to over-accumulation of ethylene. PBZ1 expression level is significantly induced in response to pathogen attacks. Following pathogen inoculation, the ACS2 levels and ethylene contents in ACS2-overexpression lines are significantly up-regulated, resulting in enhanced resistance to ShB. EIL1, the core component of the rice ethylene signaling pathway which regulates the expression of ethylene-responsive genes, positively regulating rice resistance to ShB. The SA signaling pathway in rice has two branches, one is the same NPR1-mediated pathway while the other is regulated by WRKY45. WRKY45 overexpression rice plants results in resistance to blast disease and leaf blight disease but has no positive effects (OPE) on ShB resistance. The function of NPR1 in the rice-ShB interaction remains unknown. Constitutive expression of transcription factor WRKY30 promotes JA accumulation and PR gene expression to increase ShB resistance in rice. JA positively regulates rice resistance to ShB. BR is a negative regulator of rice resistance to ShB. WRKY53 directly activates the expression of SWEET2a, a negative regulator of rice resistance to ShB, to confer susceptibility to ShB. SLR1 is the only DELLA protein in rice that inhibits GA signaling and its mutation significantly increases the disease susceptibility to leaf blight disease. ABA is primarily a negative regulator of immunity that regulates rice resistance to leaf blight disease and blast disease. The effects of SLs and CTK on the ShB process are not clear and require further investigation.

**Figure 3:**
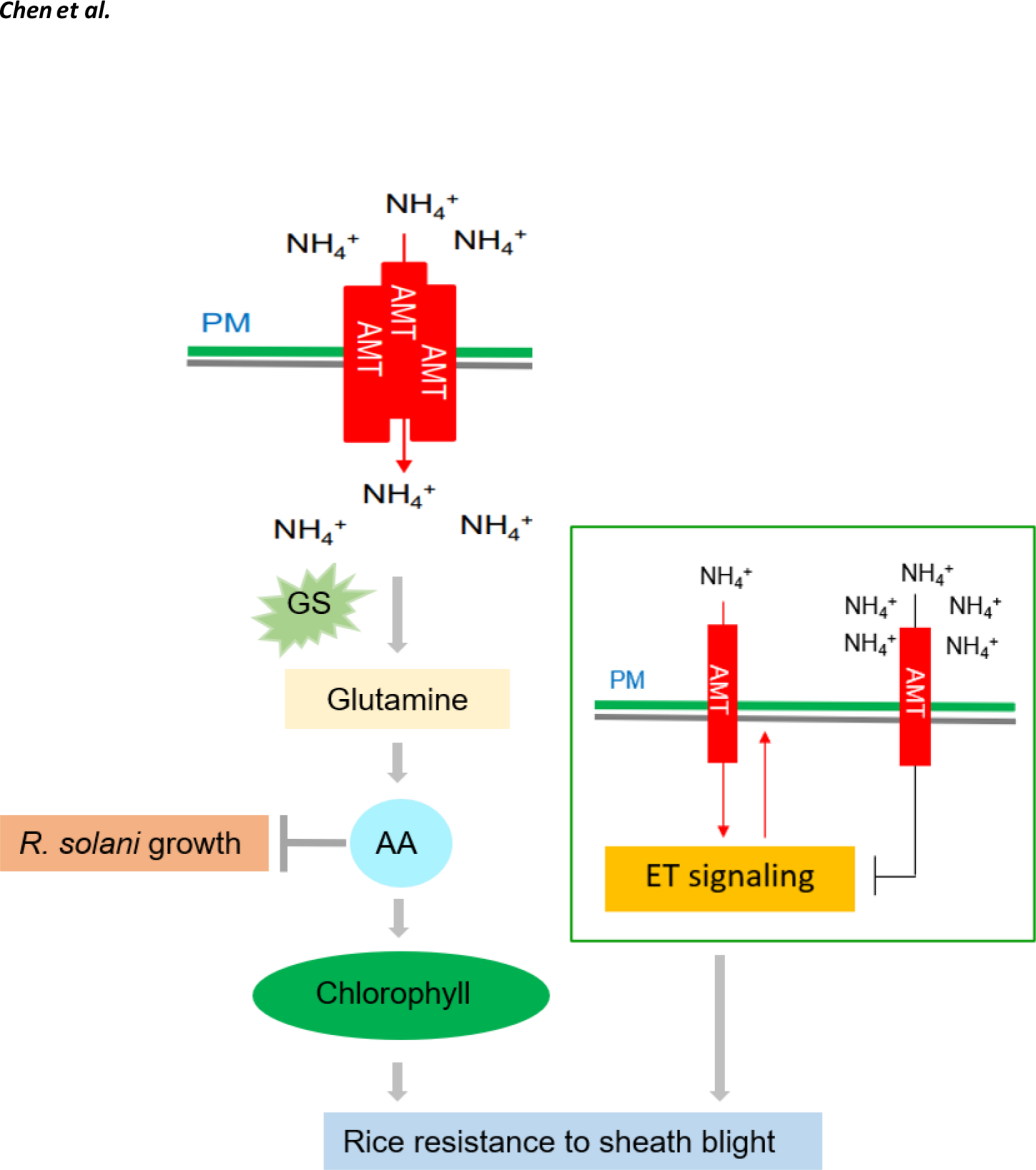
Effects of nitrogen on rice ShB. The rice ammonium transporter AMT1;1 positively regulates rice resistance to ShB. This phenomenon is caused not by ammonium itself, but by N-derived metabolites. AMT1;1 enhances the resistance of rice to ShB by promoting the accumulation of N metabolites, such as amino acids and chlorophyll, and activating the downstream ETH signaling pathway. Amino acid (AA) accumulation can inhibit R. solani and promote chlorophyll synthesis, which is a positive regulator of rice ShB. A low concentration of NH+4 activates the ETH signal through AMT and a high concentration of NH+4 inhibits the ETH signal. ETH signaling positively regulates ShB resistance and NH+4 uptake, suggesting that ETH signaling acts downstream of AMT and that NH+4 uptake is also under feedback control.

We apologize for this error and this does not change the scientific conclusions of the article in any way. The original article has been updated.

